# Raising the bar: translating global guidelines to achieve local policy on cholera elimination in Zambia—a qualitative case study

**DOI:** 10.1186/s12961-026-01464-7

**Published:** 2026-09-10

**Authors:** Chanda Mwamba, Tikulirekuti Banda, Mazyanga Mazaba, Victor Mukonka, Kennedy Malama, Esther Hamweemba, Mainza Syulikwa, Roma Chilengi, Anjali Sharma, Christine Fahim, Robert Marten, Sonam Yangchen, Sharon Straus, The RAISE Project

**Affiliations:** 1 Centre for Infectious Disease Research in Zambia, Lusaka, Zambia; 2 Zambia National Public Health Institute, Lusaka, Zambia; 3 Ministry of Health, Lusaka, Zambia; 4 Knowledge Translation Program, Unity Health Toronto, Toronto, Canada; 5 Alliance of Health Policy and Systems Research, WHO, Geneva, Switzerland

**Keywords:** Knowledge-to-action, Cholera elimination, Multisectoral approaches, SSA, Zambia

## Abstract

**Background:**

The global roadmap to eliminating cholera by 2030 relies on early detection and response, vaccination in hotspots and effective partnerships. In sub-Saharan Africa, where 60% of the 2.8 million annual cases occur, cholera elimination requires a multisectoral response. To accelerate progress, improved understanding of how to translate a multisectoral approach to eliminate cholera is needed.

**Methods:**

We evaluated implementation of Zambia’s Multisectoral Cholera Elimination Plan (MCEP) using six phases of Graham’s Knowledge-to-Action (KTA) framework (adaption, assessing barriers, implementation, monitoring, evaluation and sustaining knowledge use). We reviewed the MCEP to assess the policy environment for supporting implementation and triangulated information from 24 key informant interviews with policy-makers and implementers, 12 in-depth interviews with cholera survivors and focus group discussions with 28 community-based neighbourhood health committee members. Thematic analysis was used to analyse the data. We present our findings under each KTA phase.

**Results:**

The MCEP prioritizes actions in hotspots, and vaccinations through a multisectoral governance and coordination structure. The appointment of a coordinator and formation of multisectoral technical working groups (TWGs) have improved dissemination, coordination and the case for additional investments in laboratory capacity, cholera vaccines and infrastructure for water, sanitation, hygiene and decentralized solid waste management. However, slow institutionalization of plans, weak coordination, inadequate funding, poor infrastructure and the coronavirus disease 2019 (COVID-19) pandemic has led to fragmented implementation. The sustainability of the MCEP is not assured without: (1) comprehensive advocacy; (2) resource mobilization (international and national budget commitment); (3) monitoring and evaluation (M&E) of progress; and (4) information synthesis and dissemination for end users.

**Conclusions:**

A multisectoral approach can help gain adequate momentum and efficiencies to eliminate cholera in Zambia, provided stakeholders are fully committed and funding is allocated to support plans. An adaptive collaborative strategy within national guidelines and policies that includes target communities is needed to support local translation, adaptation and concerted efforts to eliminate cholera.

## Background

Globally, an estimated 1.3 billion people are at risk of cholera infection, resulting in approximately 2.8 million annual cases and 91,000 deaths [1]. Approximately 60% of these annual cholera cases occur in sub-Saharan Africa (SSA) [2]. Cholera emergencies devastate Zambia and its neighbouring countries owing to the loss of lives and economic costs, disrupting an already overburdened health system [3]. The Government of the Republic of Zambia (GRZ) has committed to eliminating cholera in Zambia by 2025, 5 years ahead of the global target set by the Global Task Force on Cholera Control (GTFCC) [2].

The 2019–2025 Zambia Multisectoral Cholera Elimination Plan (MCEP) aims to reduce cholera-related mortality by 90% as per the GTFCC initiative, “Ending Cholera: The Roadmap to 2030” [3]. The GRZ has adapted the GTFCC’s strategies to eliminate cholera by (1) containing outbreaks through early detection and rapid emergency response on the basis of robust community engagement, surveillance and strong health systems; (2) using multisectoral approaches to provide effective interventions such as water, sanitation and hygiene (WASH) and oral cholera vaccines (OCV) in cholera-prone areas; and (3) establishing effective mechanisms of coordination for technical support, advocacy, resource mobilization and partnership at local and global levels [2–4]. While countries in SSA are well-versed in multisectoral responses during emergencies, more insight is needed on translating a multisectoral approach to cholera prevention and elimination [5–8].

We aim to study the translation of evidence-based international guidelines and national plans into multisectoral practice by applying the Knowledge-to-Action (KTA) framework to the Zambia MCEP case [8, 9]. Based on the theory of planned action and social constructivism [9, 10], the KTA framework has been widely used to support evidence-based implementation in healthcare settings and WASH programmes [8, 10–12]. The framework considers both knowledge creation and its enactment in the action cycle through eight phases, namely: (1) identification of problem, (2) selection of relevant knowledge, (3) adaptation of identified knowledge to local context, (4) assessing barriers to acting on relevant knowledge, (5) implementing interventions to promote knowledge use, (6) monitoring knowledge use, (7) evaluating programme outcomes and (8) sustaining knowledge use. Examining these processes in MCEP implementation can help synchronize knowledge and practice in cholera elimination and accelerate progress towards GTFCC goals to end cholera by 2030 [2–5].

## Methods

### Study design

We conducted a qualitative case study design [13] composed of a review of the MCEP, in-depth interviews, focus group discussions and key informant interviews [14, 15]. The case study design allowed for an in-depth examination of MCEP, while the interviews explored its implementation from stakeholder perspectives using different qualitative methods. We used in-depth interviews to explore individual experiences, perspectives, and personal narratives; key informant interviews to obtain insights from experts or individuals with specific knowledge and expertise relevant to the MCEP; and focus group discussions to capture insights into shared perspectives, consensus or divergence of opinions among participants [15]. We used phases 3–8 of the KTA framework [8, 10–12] as listed above to help us further explore, structure and reflect on findings regarding the processes of adapting and implementing the MCEP in Zambia.

### Study setting and participants

From 1977 to 2018, Zambia experienced 29 cholera emergencies, with recorded cases ranging from 14 to 13 500, and case fatality rates ranging between 0.5% and 9.3% [16]. These emergencies occurred in periurban areas and fishing camps sharing waters with the Democratic Republic of Congo, Mozambique, Tanzania and Zimbabwe [17]. As typical of unplanned settlements and of fishing camps worldwide, poor sanitation, water, drainage and solid waste management systems characterize these areas [18]. Rapid urbanization further strains these systems, and high cross-border movements with cholera-prone countries complicates policy and implementation for cholera control [17, 19].

We conducted the study in two known cholera hotspots: (1) Mpulungu District and (2) Lusaka District [17]. These sites experienced cholera outbreaks between 2017 and 2019 [17–20], and their selection provides the opportunity to assess differences in MCEP implementation in the swampy settings of Mpulungu and the densely populated periurban settings in Lusaka [20].

### Sampling methods

We used purposeful and snowball sampling [21] to identify and select stakeholders involved with the Zambian MCEP, cholera survivors and community healthcare workers in the two study districts. These sampling methods captured multisectoral diversity such as local government, health, water, sanitation, and environmental protection; non-governmental and multi-lateral partners; and urban–rural areas.

### Data collection method

We reviewed the MCEP and conducted in-depth interviews, focus group discussions and key informant interviews from April 2020 to May 2021.

#### MCEP document review

We (A.S. and T.B.) independently reviewed the MCEP [22] for contextual and historical information, evidence of knowledge contextualization, including identification and mitigation of foreseeable challenges and risks, monitoring, evaluation and timelines planned for implementation. We considered sections of the MCEP applicable to our six KTA phases, taking notes in Microsoft Word. We then read the entire MCEP to further contextualize sections, draw conclusions and triangulate information received through key informant interviews, in-depth interviews and focus group discussions. We retained conclusions on which both researchers agreed.

#### In-depth interviews, key informant interviews and focus group discussions

Following authorization from the healthcare facility in-charges, we shortlisted adult cholera survivors registered in the environmental health technologist (EHT) registers with telephone numbers. We contacted survivors to confirm that they had cholera and were willing to participate in the study. We conducted in-depth interviews at two first-level hospitals in Lusaka and at one rural and one urban clinic in the Mpulungu District [23]. Typically, first-level hospitals are referral full-service facilities serving 20,000–80,000 people, while urban and rural clinics serve approximately 50,000 and 10,000 people, respectively [23].

Stakeholders involved in the October 2017 to May 2018 cholera outbreak were recruited. The outbreak affected 7 of the 10 provinces in Zambia, with 5905 suspected cases and 98 deaths [24]. These stakeholders included: health facility in-charges and environmental health officers at the facility level; officials from the district and provincial office; and stakeholders from various non-governmental organizations (NGOs) and multilateral organizations. Key informants were recruited from an MCEP stakeholders meeting in July 2020. Of the 24 key informant interviews, the last 11 were conducted via phone due to coronavirus disease 2019 (COVID-19)-related concerns. In-person data collection was carried out in private rooms in locations selected by the participant. In-depth and key informant interviews lasted 30–50 min.

Focus group discussions comprising six to eight participants per group were conducted with neighbourhood health committee members. We issued open invitations to neighbourhood health committee members and explained the study to them. Discussions were held at a location within the health facilities using a combination of English and the local language (Nyanja and Bemba). Focus group discussions lasted 40–60 min.

Interviewers utilized guides with open-ended questions and created field notes daily.

### Data management and analysis

All audio-recordings, made with participant consent in Nyanja and Bemba, were directly translated into English. Discrepancies between transcripts and audio-recordings were addressed by referencing field notes and team discussions. Audio-files and transcripts were stored in password-protected, encrypted study computers.

A thematic analysis approach using matrix coding was applied to categorize and synthesize data [24]. The five-member team compiled deductive codes informed by the interview guides and the KTA framework. The team read the scripts to familiarize themselves and identify codes inductively. Data were organized under code labels using an iterative coding process. The authors (A.S., C.M. and T.B.) checked the codes and their applications and grouped them into themes and subthemes with quotes. KTA domains further guided the analysis and interpretation of the thematic summaries to include contextualization of knowledge, barriers and facilitators, monitoring knowledge use, evaluating outcomes and sustainability of knowledge use.

## Results

The study included women and men aged ≥ 18 years directly involved in MCEP formulation and implementation at national, provincial, district and community levels; cholera survivors; and community health workers from cholera hotspots. A total of 12 in-depth interviews with cholera survivors in Lusaka and Mpulungu districts were conducted (See Table 1 for sociodemographic information). Furthermore, four focus group discussions were conducted with 28 community healthcare workers (neighbourhood health committee members). Additionally, 24 key informant interviews were conducted involving health facility in-charges (*n* = 3); environmental health officers at the health facility level (*n* = 6); officials from district, provincial, and national government offices (*n* = 11); and stakeholders from NGOs and a multilateral organization (*n* = 4).

**Table 1:** Sociodemographic information of cholera survivors participating in in-depth interviews

District	Number
Lusaka	6
Mpulungu	6
Sex	
Male	6
Female	6
Age range, years	
30–40	5
41–50	5
50+	2
Education level	
Secondary	7
Tertiary	5

We present findings according to six KTA phases: (i) adaptation of knowledge to local contexts, (ii) assessment of barriers to knowledge use, (iii) implementation, (iv) monitoring of knowledge use, (v) evaluation of outcomes and (vi) sustainability of knowledge use. The national cholera elimination policy environment context is detailed in the review of the MCEP, while the in-depth interviews, key informant interviews and focus group discussions illustrate the knowledge translation, adaptation and application. An iterative process of comparison and triangulation of all data sources informed interpretation and implications. Table 2 summarizes the barriers and facilitators to the adaptation and implementation of MCEP by location.

**Table 2:** Barriers and facilitators to operationalize MCEP

Adaptation capacity						
Location	Human, economic and infrastructural resources	Knowledge translation and transfer	Stakeholder coordination strengthening	Community engagement and reach of MCEP	Dissemination and enforcement of policies	Political will
Lusaka	MCEP delivered the necessary framework to plan, fund and track implementation Inadequate funding of MCEP activities at national level Fragmented resourcing affecting funding and coordination of resources Resource mobilization key pillar of MCEP Insufficient resources to manage infrastructural developments for provision of public services including water, sanitation, drainage and waste collection in densely populated compounds Dedicated structures to implement cholera programmes at provincial, district and facility levels Need for continuous skills development to solve adaptation challenges Cholera elimination included in health development plans embedded in Zambia’s 7th National Development Plan Investments and response are biomedical with funding accelerated during an outbreak Funding priority on COVID-19 may affect funding of MCEP activities Financing and implementation coordination heavily dependent on government ministries who lack funding within their wider portfolio to prioritize operationalizing policies	Health leadership teams at national and provincial level had knowledge of MCEP Gap with evidence synthesis to translate knowledge NHRA improving knowledge translation capacity Non-governmental and multilateral actors had different ideas of the overall MCEP strategy Highlighted need for skills training for NHCs and other community health workers to support implementation of oral cholera vaccines	All cognizant of the need for strong coordination, accountability, commitment and ownership Challenges with cross-sectoral coordination owing to diverse responsibilities and competing priorities Multisectoral partners not visible and networking largely dependent on Ministry of Health Appointment of the national coordinator and formation of technical working groups improved cross sectoral coordination Emergency preparedness plans included at each level; promotes coordination to reach hotspots and enhanced efficiency and effectiveness	Community engagement key to cholera elimination Community engagement tool for elimination of stigma	Call for a proactive approach such as the dissemination and enforcement strategies used for COVID-19 Adaption linked with infrastructural developments. Enforcement hindered by lack of planned sewer networks, waste disposal systems and piped water	Cholera elimination linked to county’s national development Political will necessary to promote policy decisions and funding initiatives Political will demonstrated by initiatives such as establishment of MCEP structures and the ZNPHI Concerns about political will based on limited government funding to WASH initiatives to proactively manage cholera risk
Mpulungu	Few community health workers overseeing large catchment areas Financial resources lacking for infrastructural development, continuous skills training and sustainable community sensitisations Adaptation linked with economic activities to reduce disease risk and vulnerability	NGO actors and implementers at facility level described cholera response activities with limited knowledge on MCEP	Good coordination at district level through the integrated development master plan Good coordination among government ministries and resource sharing for implementation of programmes Room to strengthen coordination between the national and district health teams in provinces outside Lusaka and with other stakeholders	Community members aware of reactive measures implemented in hotspots during an outbreak; these stopped when an outbreak ended Involvement of chiefs in community engagement strategies key Community-led initiatives support sustainable enforcement of measures	NHC members outside Lusaka recommended a bottom-up approach supported by policy to promote implementation and enforcement The local authority in collaboration with NGOs and community members implemented the community-led total sanitation initiatives Village headmen key to foster community engagement and enforcement	Call for improved political will to address economic/livelihood concerns, infrastructural development and funding for long-term cholera elimination

### Adaptation of knowledge to local contexts

**Fig. 1:**
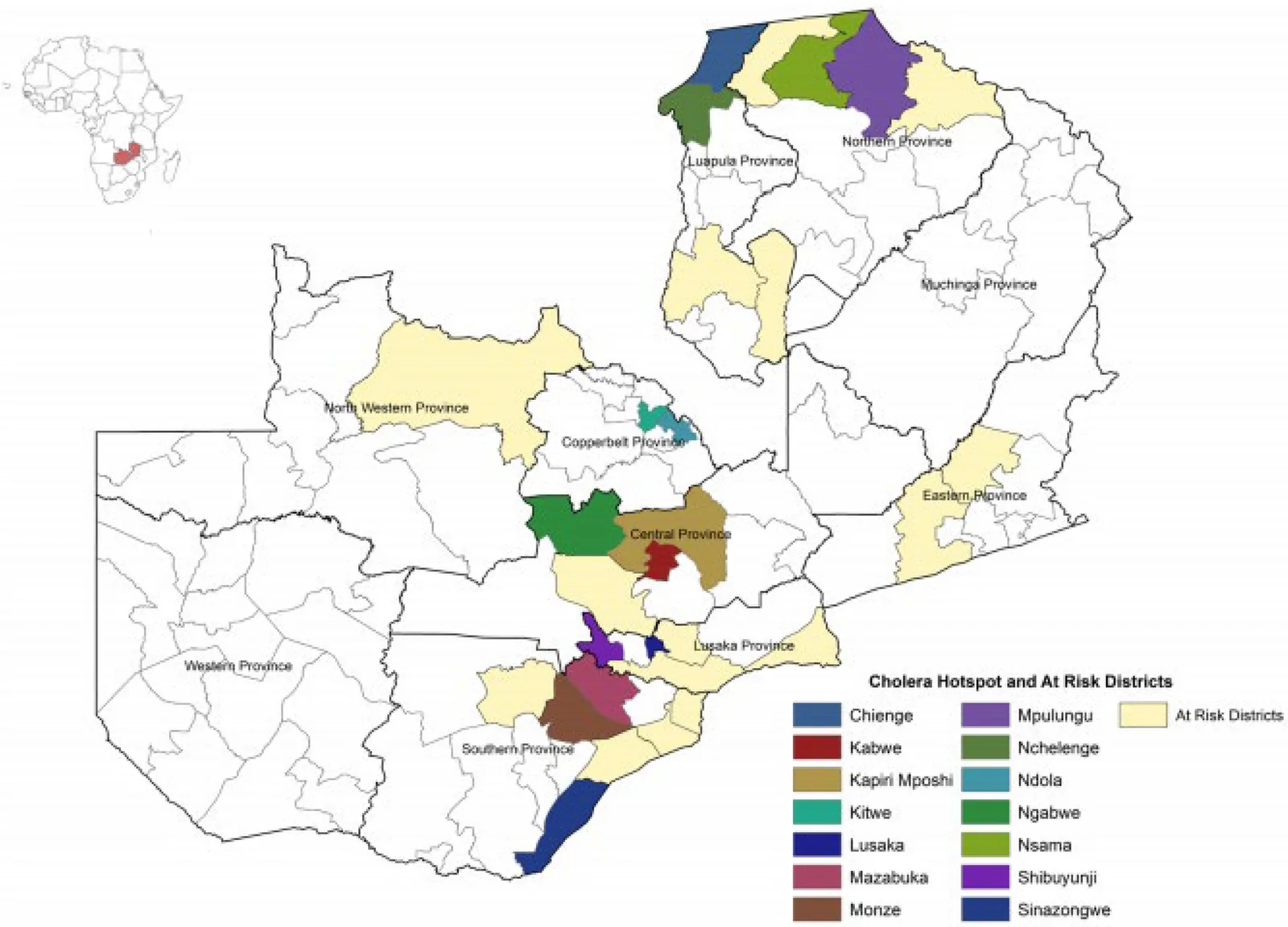
MCEP cholera hotspots and at-risk districts [17]

The document review revealed contextualization of the MCEP, which strategically targets 12 cholera hotspots for OCV campaigns to gain quick results and public confidence before implementing bigger infrastructure and behaviour change projects. These hotspots were identified through epidemiological, geographical and contextual analyses to include areas with recurrent outbreaks, poor WASH services, transit points, presence of slums, flooding, fishing camps and influx of refugees. The MCEP implementation targets a second set of high-risk areas with a history of cholera outbreaks, poor WASH facilities (< 50%) and crowding in both urban and rural areas for improved access to basic WASH facilities and solid waste management (Fig. 1).

**Fig. 2:**
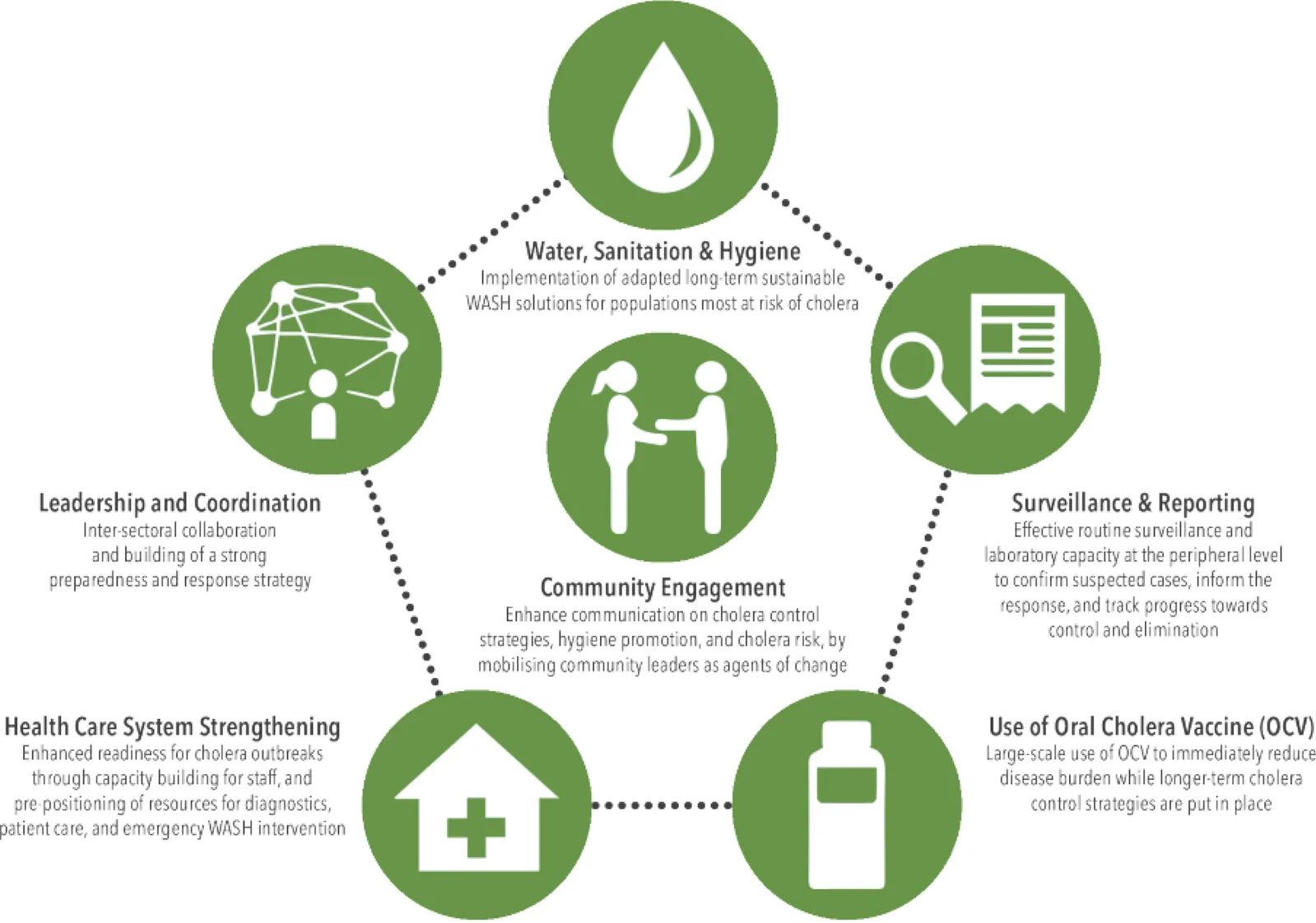
MCEP multisectoral interventions to end cholera [2] (Adapted from GTFCC)

The decentralized, multisectoral approach to cholera elimination (Fig. 2) rests on multisectoral frameworks such as Vision 2030 for Zambia, Sustainable Development Goals 2030 and 7th National Development Plan 2017–2021, as well as partnerships and cross-sectoral coordination mechanisms at different levels of government. The MCEP emphasizes importance of leadership and coordination through technical working groups (TWGs) on WASH, case management, surveillance and laboratory, community engagement and OCV. Furthermore, the plan has mobilized local and international partners to work together towards fulfilling outlined activities.

The MCEP allows for multisectoral, private–public and inclusive efforts to prevent cholera through OCV campaigns; water quality checks; improved WASH and solid waste management; and community engagement in hotspots, with particular attention to transit points, service areas (e.g. clinics, markets and restaurants), public toilets and places where people congregate (e.g. schools, workplace and churches). Collaborations between cross-border security, immigration, clinical staff and communities promote uptake of WASH and OCV in border towns. Early case detection through surveillance and effective case management aims to interrupt transmission. The plan recognizes but does not clearly address the risks posed by poor infrastructural facilities, natural catastrophes (e.g. floods or droughts), humanitarian crises and climate change. For instance, cholera survivors, neighbourhood health committee members and district officials commented on poor housing, crowding and poor road networks in Mpulungu, which can make WASH interventions challenging.

Though some key informants (KIs) indicated that cholera elimination activities were generic, others provided examples of adaptations. For instance, the revitalized community-led total sanitation (CLTS) projects in Mpulungu sensitize local communities to eliminate open defecation around lakeshores and on high grounds to prevent contaminating this primary source of drinking water for lake dwellers. The concerned local authorities (members of parliament, chiefs, council members and district line ministries), NGOs and community members also implement alternative livelihood projects and the “keep Mpulungu clean” initiative in cholera hotspots.

During focus group discussions, neighbourhood health committee members reported that adaptations in Lusaka focused on proactive strategies. This included installation of pipes and drainage systems, and burying of shallow boreholes to protect water from contamination, resulting from surface wells and poor water reticulation systems. Reactive measures after cholera outbreaks included the creation of designated rubbish collection points, the provision of flushable toilets in some compounds (highly populated unplanned settlements) and communal boreholes for water. International consulting firms, NGOs and bilateral institutions supported these improvement projects.

### Barriers and facilitators to implementation of MCEP

Contextual matters that affect translation, adaptation and implementation of policies are related to: (1) human, economic and infrastructural resources, (2) knowledge translation and transfer, (3) stakeholder coordination, (4) community engagement, (5) dissemination and enforcement and (6) political will. In this study, the second phase of the KTA framework, which focuses on assessing barriers, encompasses a broader perspective by considering both barriers and facilitators.

#### Human, economic and infrastructural resources

Key informant interviews revealed that dedicated human resources at provincial, district and facility levels have the expertise to affect cholera elimination programmes. However, national government actors also noted the need to improve and continuously develop specific skills, for example, “surveillance, case management, and WASH management”. Facility-level actors relied on volunteers for community-based implementation, who were insufficient in number. One neighbourhood health committee member in Mpulungu commented that health facilities “should find many more volunteers … We have about 1600 houses in our village and only two people to visit these houses. It is just not enough”.

There was no specific budget allocation to the MCEP, making it easy to redirect funds to the COVID pandemic instead of embedding COVID prevention in cholera elimination activities, for example. National government officials and NGO actors indicated that financing and implementation of cholera activities depended on ministries that had different priorities within their wider portfolio. Fragmented resourcing also affected the funding and coordination of resources to implement the cholera elimination agenda, leading to mistimed fund disbursement and investment in temporary measures such as water tanks for safe drinking water during cholera emergencies. As expressed by an NGO actor “Cholera is seen as health rather than a development issue” such that funds are only made available during an outbreak, which “is expensive and usurp resources meant for developmental activities”.

Though the government created the Ministry of Water Development, Sanitation and Environmental Protection, the 2020 national budget did not provide adequate funding for WASH infrastructure development, as noted by all participants. Lack of funding for infrastructure development to provide water, sanitation, drainage and waste collection services affected how communities received and perceived cholera guidelines:

We have noticed that institutionalization of the MCEP by key ministries is slow because of lack of understanding of the elimination agenda, and ministries have got different priorities. Ministries do not put the elimination agenda into context and concentrate on their ministry or organizational priorities. (National government official, key informant interview)

We are not taking advantage of evidence that is available. Policies and strategies have been put in place, but at implementation level you will find that there is no water and sanitation. (District government official, key informant interview)

While inadequately funded, some WASH infrastructure projects are underway in hotspots, including in Mpulungu and Lusaka, to improve water supply. However, cholera survivors from Mpulungu felt that poor infrastructure, specifically poor housing, crowding and poor drainage systems may challenge WASH interventions.

Community-based health workers reported that limited funding also affected availability of supplies, such as chlorine, after cholera outbreaks or protective clothing. Neighbourhood health committee members were particularly demotivated owing to lack of allowances, lack of personal protective equipment and transport challenges to conduct sensitizations and monitoring activities in communities. Additionally, OCVs were provided as one-off activities unlike other vaccines that are readily available at health facilities, such as polio vaccines.

#### Knowledge translation and transfer

National and provincial government health leadership demonstrated knowledge of MCEP strategic direction, coordination and implementation plans. NGO actors and implementers at health facilities, specifically from Mpulungu, had an overview of field activities but limited information on the MCEP, with an NGO actor in Mpulungu saying, “I am learning of that document today, but I am aware of the cholera taskforce at district and provincial level. Those are the people coordinating to end cholera”

Government, non-governmental and multilateral actors suggested different ideas of the overall MCEP strategy. For example, some government national actors indicated that the MCEP implementation strategy was largely bottom-up, with communities developing programmes and owning policies:

We interact with people; understand their problems and we try to work with them at all different levels. Our principles are to start from where people are and what they have, to organize community members to use their own resources to achieve certain goals … to reduce dependency on MOH. (National government official, key informant interview)

However, NGO actors and facility-level actors thought guidelines were top-down:

We need communities to identify their own problems and find solutions for themselves so that it is easier for them to implement solutions that come from them, unlike the current situation where they are told to follow guidelines made from elsewhere. (NGO actor, key informant interview)

#### Stakeholder coordination

All participants thought that strong coordination, accountability, commitment and ownership of the program was needed for the MCEP to achieve its desired impact. The multisectoral approach complicated the response because “Elimination involves more ministries, and more stakeholders” and coordination must ensure “that each stakeholder does their part when each is independent and cannot be dictated to”. In particular, response and coordination of the MCEP was perceived to be largely driven by the MOH, leading to variability in responsibility and dedicated resources set aside by stakeholders:

The cholera elimination plan should allow for visibility of … different stakeholders to reduce the dependency on MOH for coordination of these activities. Visibility of these other partners would make them see themselves as important partners in this process. (NGO actor, key informant interview)

#### Community engagement

Participants indicated that community engagement was key to cholera elimination. Cholera survivors and neighbourhood health committee members suggested the need for continuous education and sensitizations to sustain cholera awareness, dispel misconceptions and support hygiene standards, particularly in densely populated compounds. They noted that while “communities react fast during outbreaks and accept vaccines, behaviours are not sustained after outbreaks”. Participants stated that lack of infrastructure such as planned structures, sewer networks and piped water is an impediment to enforcement of cholera guidelines:

People cannot act on this information about hygiene. For example, people use shallow wells and when we ask them to bury these wells, they argue to say, ‘Where do you suppose we will get water after we do what you have asked us to do?’ (Neighbourhood health committee member, Mpulungu, focus group discussion)

However, not everyone valued cholera prevention interventions. For example, some people in Mpulungu vandalized communal water and sanitation structures constructed during the 2018 cholera outbreak.

During outbreaks, some people may seek “herbal remedies thinking cholera was due to witchcraft” or may not come to healthcare facilities owing to cholera-related stigma. Cholera survivors and NGO actors highlighted that community engagement could stop stigma against survivors and their families:

To make things worse, people had a lot of stigma after cholera broke out. If someone is infected with cholera, they would not want to talk to that person. It took time for people to get back to normal because organization such as Oxfam came in to support community sensitizations. That is when people started to change their mindsets. (Cholera survivor, woman, in-depth interview)

On a positive note, cholera survivors indicated that overall, when they had cholera, health facilities responded quickly and comprehensively, providing them with treatment and preventive measures in their communities:

The clinic reacted fast when we had the first case of cholera, they got my samples, and I was admitted and taken to the cholera centre. A day after I was taken to the cholera centre, they went to my community and distributed chlorine to my neighbours and sensitized them on how to keep themselves safe. (Cholera survivor, man, in-depth interview)

#### Dissemination and enforcement of policy

Shortfalls in health communications notwithstanding, participants noted some effective strategies for uptake of policy. For example, the involvement of chiefs in community engagement strategies within cholera hot spots in Mpulungu may have contributed to behaviour change among communities. In Lusaka, dissemination of messages done mainly through door-to-door sensitizations, drama shows, brochures, radio and television messages were perceived as effective.

I will go back to 2018/2019, the time we had cholera and the MOH came and trained, so we had an opportunity to go in the community. The chiefs called for a meeting, after that we started teaching the community. (Neighbourhood health committee member, Mpulungu, focus group discussion)

We used drama groups because they are good in working with people. Also radios and television. Even churches can be used to communicate because there are many. (Neighbourhood health committee member, Lusaka, focus group discussion)

Participants indicated that incorporating policy framework and community engagement with risk communication approaches is important for information dissemination and required enforcement. They suggested drawing lessons from the COVID-19 policy and enforcement response.

We are wearing masks because that’s what guidelines say, we are washing and sanitizing our hands … Surely, we can learn from COVID-19 – how policies were quickly there, and the way communities were made to comply … (Neighbourhood health committee member, Mpulungu, focus group discussion)

#### Political will

All participants mentioned that cholera elimination has value to households, communities and health systems, releasing opportunity and monetary costs to contribute to national development. However, some expressed concern at the lack of political will to make policy and budgetary allocation to address economic-livelihood concerns as well as WASH and infrastructural development for sustainable cholera elimination:

This is time to walk the talk if the government wants to bring any change and see that cholera is eliminated (NGO actor, key informant interview)

We have government will to make sure that all planned activities are funded and the commitment and passion from the people that are involved to make sure that these goals are achieved (…) However, we are not yet there so we need to build this spirit where all involved ministries can ensure that in their budget line there should be a talk of cholera somewhere. For now, we have no specific resources for cholera. (National government official, key informant interview)

KIs noted government commitment to prevent rapid spread and deaths during outbreaks. Government KIs pointed out that, “Cholera is a development issue dependent on WASH with huge effects on national development”, but acknowledged that pre-emptive actions are biomedical (OCV) and that efforts wane post-cholera outbreaks. They added that political and economic interests may sometimes interfere with implementation, for example, when markets are allowed to remain open despite being identified as reservoirs for cholera during outbreaks.

### Implementation

Despite the predominant barriers reported by stakeholders, implementation has progressed in Zambia. Key MCEP actors appointed the national cholera elimination coordinator and formed TWGs to improve cross-sectoral coordination, which allowed the MCEP secretariat to actively lobby for external, governmental and community funding. Zambia is among the first four countries to receive GTFCC funds “in recognition of the effort made towards the elimination agenda”. The Leadership team continues to advocate for inclusion of the MCEP in the national budget, engaging members of parliament, especially in the committee of economy. Additionally, each level of the health system now has emergency preparedness plans that have been used to “advocate for strengthened collaboration to manage cholera outbreaks” for efficient programme implementation:

We also developed the integrated development plan, which is a master plan for the district; information in this document helps to show us which areas are prone to various issues … We liaise with other government officials … that way we use the same vehicle to go and do different activities in a similar ward. We are working with coordination in a multi sectorial approach. (District government official, key informant interview)

Community-led approaches, such as the CLTS, provide a platform for communities to own and participate in programmes and adapt behaviours. These community approaches involve different community structures, such as chiefs and area counsellors, government, and NGO institutions. District actors provide training on specifications for a basic, clean and safe toilet with hand washing facilities, while the community builds toilets to address problems such as open defecation.

### Monitoring of knowledge use

The MCEP provides an elimination plan, monitoring framework, implementation timeline and a list of operations research, which most KIs thought useful to “plan, fund, and track implementation”. KIs emphasized the need to have strong M&E to ensure timely implementation and account for donor funds:

If you account for the resources we allocate to the fight against cholera then many players or stakeholders will come on board to assist us but if not, then the players will not play their part well. (NGO actor, key informant interview)

The monitoring framework has indicators for strategic use that measures the attainment of cholera elimination, including reduced case fatality rate, early detection and prevention of local cholera transmission, coordination for technical support, and finances and partnership for program implementation.

Programme indicators for each of the monitoring framework pillars have instrumental use as they measure milestones from start-up (e.g. plan developed, funds secured and staff trained) and lead into strategic indicators:

It also depends how committed the committee you have put in place is to ensure the monitoring and coordination of the elimination plan is coherent and robust. Different stakeholders must be brought on board and be made accountable, to what extent that is done will have an impact on whether you are achieving or not. (Multilateral organization actor, key informant interview)

The leadership and coordination of the MCEP include various stakeholders as a representation of sectors, technical competence and geographical representations. The surveillance pillar measures community reporting, case investigation and the capacity for cholera and antimicrobial resistance laboratory testing. Though required weekly, district players made monthly reports to the province, who sent consolidated reports every quarter to the national level.

Other than measuring presence of good practices and required training and supplies, case management also requires additional research to map and place laboratories and cholera treatment units in hotspots. OCV monitoring similarly comes with an injunction to work with GTFCC, Gavi, the Vaccine Alliance and other partners to secure sufficient vaccines and ensure their use in cross-border locations.

Additionally, WASH is measured by uptake of WASH facilities/provisions and their use, for example, proportion of households with basic sanitation and hygiene facilities, and proportion of households not practising open defecation. Monitoring indicators measure achievements of plans to expand laboratory and environmental health surveillance systems, while OCV, case management, risk communication and community engagement operate within existing systems.

### Evaluation of outcomes

Monitoring of indicators is being used strategically to evaluate outcomes. The main goal is the absence of evidence of community transmission of confirmed cholera cases for 3 years in a row by 2025. Zambia has not had an outbreak since 2019, which national key informants attribute to high uptake of OCV in hotspot areas during and after the 2017–2018 outbreak. Those hesitant about the cholera vaccine received information and education, as a result, the national key informants reported “from the time people got vaccinated, we have not recorded a single case of cholera”. Others added that the absence of cholera cases “could be because we did the vaccination activity, we have intensified cleaning of the district and people now know how to use chlorine. So, I think it was due to preventive measures, information and for some reduced poverty”.

### Sustainability

KIs revealed the gap in evidence synthesis to translate knowledge because of limited demand and lack of user-friendly packaging for knowledge users, including policy-makers and communities:

So, there is a problem on both sides from the knowledge users who are the policy-makers and the knowledge supply side who are the researchers due to inadequate interaction … There has not been enough sensitization in terms of knowledge translation. On the other hand, the producers of knowledge, the researchers themselves have not been as forthcoming in terms of packaging that information in a form that knowledge users can utilize. (National government official, key informant interview)

Though the National Health Research Authority (NHRA) is responsible for knowledge synthesis, they were only included within TWGs after the MCEP was developed. Being fairly new, the NHRA is establishing systems and providing policy-to-practice courses to improve the nation’s and their own capacity for knowledge synthesis and translation. Furthermore, government actors asked for more research such as identifying the primary cause of cholera outbreaks to design appropriate interventions. For example, a district actor remarked that while interventions aim to provide clean water and individual toilets in Lusaka, they would not be effective if a cholera outbreak was due to improper food handling practices. Though the MCEP aimed to conduct operation research, no funds have been earmarked for locally determined and led research.

Those who knew the MCEP felt safe when implementing cholera elimination initiatives. Midcourse changes described in the implementation section could address the barriers presented in this paper to achieve cholera elimination. However, KIs urged for more momentum and intense actions, such as increased financing for the MCEP, capacity building of staff, and improved knowledge translation and transfer to meet all targets set for 2025 and to ensure the continuous implementation needed to eliminate community transmission of cholera and reduce cholera-related deaths by 90%.

## Discussion

Our study evaluated MCEP implementation using the KTA to assess knowledge collection and translation. Our document review highlights the multisectoral governance structure with varied roles for cross-disciplinary organizations and actors. Though supportive, actions of these organizations were filtered through disparate sector objectives owing to limited funding, aims and broader organizational culture and interests. Institutionalization of collaborative transdisciplinary approaches to respond to different settings, inclusiveness and iterative knowledge translation are needed [8, 10, 25, 26]. Integrating MCEP policies into organizational policies, planning and practice could be one strategy of harnessing fragmented efforts by relevant actors [8, 10, 25, 26].

Adding MCEP policies into various organizations’ policies alone may not address the highlighted challenges. Having cross-sector champions, acknowledging the different actors’ value, strengths, and capacity, and balancing the MCEP implementation requirements with their specific organizational interests could optimize MCEP KTA [26, 27]. Creating these structures has value in promoting consistent stakeholder engagement to facilitate collaborative involvement in dynamic knowledge transfer processes [28]. Incorporating community knowledge, specifying the research framework and having a robust decision-making framework would ensure that context is given due consideration in translating MCEP knowledge into action [29]. These findings are similar to a study conducted in Canada, which found that for knowledge translation researchers, developing processes to assist community-based organizations adapt research findings to local circumstances is the most helpful way to enhance decision-making processes in knowledge translation [30]. The study also found that knowledge producers were unable to convey or translate findings to usable products for policy programme development. For instance, while many scholars in various fields have been focusing on utilizing knowledge, they have paid little attention to content or knowledge that should be translated and the ways it could be collected, organized, delivered understood and eventually utilized and synthesized [30, 31]. An adaptative collaborative strategy within the national guideline to support local translation, adaptation and concerted efforts can thus be cardinal in bridging this existing gap.

The creation of the NHRA is meant to coordinate research and bridge the knowledge translation gap in national health objectives [32]. The NRHA has made efforts since its inception to strengthen synergies between researchers, policy-makers and other stakeholders. Herein, the NHRA acknowledged their inconsistent involvement in cholera knowledge translation and missed opportunities to incorporate structured translational research processes to tailor information for policy-makers [11, 12, 31]. Enhanced linkages between NHRA, health policy-makers and implementors, along with consistent and sufficient government support, are essential to fully valuing the role of knowledge translation in the implementation of programmes [11, 12, 31, 32]. These findings are similar to a study conducted in Zambia, which highlighted the value of strategic leadership to manage linkages between policy-makers and researchers while building capacities in knowledge synthesis, communication and brokering [33]. Lessons from the study underscored the importance of developing coherent national/institutional KT strategies that are embedded within KT structures across various governance levels. These strategies can be embedded into policy development and decision-making processes.

The MCEP provided the national framework to mobilize funding for cholera elimination programmes. Although Zambia was one of the first countries to receive external funding, cholera elimination as planned requires careful consideration of sustainable funding, including within the local health-related budget lines for infrastructure development, WASH efforts and vaccinations [2, 3]. Zimbabwe and Ethiopia similarly have insufficient funds to implement the MCEP. The GTFCC has provided technical and financial support to implement Zimbabwe’s 2018–2028 MCEP, with limited evidence indicating domestic funding of the plan [34]. Similarly, 50% of Ethiopia’s cholera emergency preparedness response plan has existing funding mechanism indicating a 50% gap in funding [35]. Global attention and diverted resources to COVID-19 may affect resource mobilization efforts and thus deliberate national action to maintain progress in cholera elimination is required, especially as COVID-19 burdens national health systems [3, 32].

Weak knowledge of the MCEP demonstrated by participants farther from the MCEP national leadership team and the Lusaka District could inevitably be a result of the centralized MCEP approach [34–37]. MCEP implementers at facility and community levels wanted to be more informed and involved to support context-specific adaptation and translate findings to usable products for their communities [30, 37–39]. Contextual factors determine the suitability, impact, uptake and sustainability of an intervention in a given setting or population, and this is best achieved through community engagement [37, 39–41]. Community engagement allows for inclusion of the community’s knowledge, facilitates feedback systems on achievements and highlights challenges and recommendations to improve interventions. Community involvement needs to be “actively” incorporated in structuring the implementation of MCEP strategies, as knowledge translation may not inherently integrate community knowledge, contexts and interests [30, 34–38].

Given the lack of sustained M&E of the MCEP, we are unable to conclude with certainty on its sustainability. Program M&E clarifies tenets of sustainability, barriers and facilitators for program improvements [12, 40]. A study conducted in Kenya found that M&E helps project managers keep track of project implementation and allows for improved resource allocation and efficiency [41]. M&E also provides decision-makers with a strategy to plan for project sustainability and guidance for future endeavours [41]. Sustainability is key to stakeholders, who need to be involved throughout the project and program cycles. Hence, the lack of the MCEP M&E could lead to ineffective structuring of program implementation and misaligned investment focus [12, 41, 42]. In this regard, there is need to strengthen the MCEP M&E to inform the different pillars of the program, including knowledge translation and implementation.

### Limitations

We did not have an objective measurement of accomplishments through observations or document verification, relying on retrospective document review and stakeholder accounts. However, by engaging with various stakeholders and triangulating data from the multiple sources and perspectives, we aimed to provide a well-rounded assessment of the program’s effectiveness and contextual factors influencing its outcomes. While we were able to provide a breadth of information confirmed by a variety of sources, we were unable to explore the roots of persistent problems owing to COVID-19 restrictions and demand of participant time. Addressing persistent problems will require more in-depth studies and methods to reach consensus on politically charged issues.

## Conclusions

MCEP implementation has focused on early detection, rapid emergency response and OCV implementation in cholera hotspots. However, mechanisms of coordination have faltered without adequate funding for cross-disciplinary mainstreaming of the cholera elimination programme into policies, budgeted annual workplans and implementation among stakeholders. Budgets must include costs for human resources, infrastructure, logistics and frequent scheduled coordinating meetings to discuss the current implementation status, evaluation of results and adaptation of strategies according to lessons learned. To eradicate cholera, national entities should synthesize, translate and adapt policies and plans into implementation practices. This should be carried out using the bottom-up approach through research and community involvement. At international levels, donor interest in cholera elimination is needed to move beyond the current biomedical support, particularly OCV procurement, to address in-country WASH inequalities and placement of technical and resource mobilization strategies to align with multisectoral cholera elimination plans. Nationally, strengthened political will will be vital in resource mobilization, institutionalization of the multisectoral response, use of knowledge translation processes and evaluation of programmes to support its sustainability.

## Data Availability

The field notes, the verbatim interview recording and the transcripts generated during data collection and analysis of this study are not publicly available to ensure the confidentiality and anonymity of the participating organizations and participants.

## Abbreviations

CLTS: Community-led total sanitation
EHT: Environmental health technologist
GRZ: Government of the Republic of Zambia
GTFCC: Global Task Force on Cholera Control
KTA: Knowledge-to-action
MCEP: Multisectoral Cholera Elimination Plan
M&E: Monitoring and evaluation
NGO: Non-governmental organization
OCV: Oral cholera vaccine
SSA: Sub-Saharan Africa
TWG: Technical working groups
UNZABREC: University of Zambia Biomedical Research Committee
WASH: Water, sanitation, and hygiene
WHO: World Health Organization
ZNPHI: The Zambia National Public Health Institute
